# Comparative Physiology of the Brain and Comparative Psychology

**Published:** 1901-12

**Authors:** 


### Reviews of Books.

**Comparative Physiology of the Brain and Comparative Psychology.** By JACQUES LOEB, M.D. Pp. x., 309. London: John Murray. 1901.

Professor Loeb might have chosen a more satisfactory title for a work which contains much original and independent research. But if some of those who turn to this book for a well-balanced exposition of what is known of the comparative physiology of the brain may find their expectations only partially realised, and if some who seek information on comparative psychology may find that the area of the subject touched on is limited, still both classes of enquirers will find much of interest and value in the account which Professor Loeb here gives of his own and cognate investigations and experiments, and in the conclusions which he feels justified in drawing from them.

Starting with a consideration of reflexes, Professor Loeb enquires what part the central ganglia play in their production. He is of opinion that we must deprive them of all specific significance, not only in the case of simple reflexes and instincts, but also in that of spontaneous movements. Their importance lies in the fact that they, in common with other nerve structures, possess in an enhanced degree that irritability and conductibility which is a characteristic of all protoplasm. He seems at times to deny to them any distinctive *rôle* in the process of co-ordination; but what he is really anxious to eliminate from scientific exposition is any metaphysical power of co-ordination, and to show that seemingly purposive responses are in the lower animals and in some plants effected without the intervention of a nervous system. He lays stress on the part played by ions in the tissues and the liquids in which they are bathed, and regards many of the tropisms, which lie at the base of a large number of purposive movements, as sufficiently explicable in chemical terms.

The earlier chapters deal with the lower organisms in which the nervous system is little differentiated. Passing to higher animals, Professor Loeb lays stress on the fact that the central nervous system is essentially a connected segmental system, and he considers how far the brain, or in the invertebrates the supra-œsophageal ganglion, or its analogue, is a supreme co-ordinating centre. To a large degree his tendency is to deny to it any such office. At the same time he does not deny to it all regulative influence on the lower centres. The fundamental fact of comparative psychology is, according to his view, what he terms the activity of the associative memory, which in vertebrates he regards as having its seat in the cerebrum. He is here putting in other words what is held to be true by not a few of those who have considered the problem of the part played by consciousness in animal life; and he might, perhaps, with profit have devoted a chapter to the anatomical connections and physiological means of influence of the seat of the "associative memory" on lower centres by means of the pyramidal tract or something analogous thereto.

The records of a large number of interesting and well-devised experiments—many of which have been published elsewhere—are here collected and will repay the careful attention of physiologists. The tone of the book is, however, a little egotistical and combative, and in some cases the author is less in antagonism with existing physiological teaching than he would lead his readers to suppose.

